# Surrogate endpoints in trials: a call for better reporting

**DOI:** 10.1186/s13063-022-06904-7

**Published:** 2022-12-12

**Authors:** Oriana Ciani, Anthony M. Manyara, An-Wen Chan, Rod S. Taylor, on behalf of the SPIRIT-SURROGATE/CONSORT-SURROGATE project group

**Affiliations:** 1 SDA Bocconi School of Management, Milan, Italy; 2 MRC/CSO Social and Public Health Sciences Unit, Institute of Health and Wellbeing, University of Glasgow, Glasgow, UK; 3 Women’s College Institute Research Institute and Department of Medicine, University of Toronto, Toronto, Canada; 4 MRC/CSO Social and Public Health Sciences Unit & Robertson Centre for Biostatistics, Institute of Health and Wellbeing, University of Glasgow, Berkeley Square, 99 Berkeley St, Glasgow, G3 7HR Scotland, UK

**Keywords:** Surrogate endpoints, Randomised trials

## Abstract

Using a surrogate endpoint as a substitute for a patient-relevant final outcome enables randomised controlled trials (RCTs) to be conducted more efficiently. However, the use of surrogates remains controversial and there is currently no guideline for the reporting of RCTs using surrogate endpoints; therefore, we seek to develop SPIRIT (Standard Protocol Items: Recommendations for Interventional Trials) and CONSORT (Consolidated Standards of Reporting Trials) extensions to improve the reporting of these trials. We would like to invite interested individuals (trial methodologists, journal editors, healthcare industry, regulators and payers, and patient/public representative groups), particularly those with experience in the use of surrogate endpoints in trials.

## Introduction

Evidence for the effectiveness of interventions should ideally come from randomised controlled trials (RCTs) that assess a patient/participant relevant final outcome (PFRO): a measurement that reflects how an individual feels, functions, or survives, such as mortality or health-related quality of life [1, 2]. However, such trials can require large sample sizes and long follow-up times and are ultimately costly [2]. One way to improve trial efficiency is the use of a surrogate endpoint that acts as proxy and predictor for the PRFO [3]. Over last 20 years, drug licensing in United States (US) and Europe has allowed the use of surrogate endpoints in the approval of new drugs and biologics, typically based on biomarkers, e.g. systolic blood pressure and/or low-density lipoprotein cholesterol for cardiovascular death, HIV viral load for development of AIDS, and tumour response for overall survival [3]. However, it is important to acknowledge the potential application of surrogates in the wider setting of non-drug trials and the use of intermediate outcomes that may lie more distally (than a biomarker) on the causal pathway and closer to a final outcome, e.g. hospice enrolment for mortality with an intervention aimed at improving end of life care [4]; fruit and vegetable consumption for cardiovascular events for a behavioural intervention designed to improve cardiovascular risk [5].

## Risks of surrogates

Despite their benefits, use of surrogate endpoints in evaluation and regulatory approval of health interventions remains highly controversial. Some drugs, approved on the basis surrogate endpoints, have failed to deliver improved PFROs and, in some cases, cause more overall harm than benefit, due to treatment effects that are not necessarily mediated through the surrogate-PFRO causal pathway [6]. A notable illustration is the diabetes drug rosiglitazone, approved by the US Food and Drug Administration (FDA) in 1999 and European Medicines Agency (EMA) in 2000 following short-term phase I–III clinical trials, showing that it improved the surrogate endpoints of blood glucose and glycosylated haemoglobin (HbA1c) [7]. However, meta-analyses of RCTs published some 10 years later together with the large RECORD trial (4447 type 2 diabetes patients followed up for 6 years) with the primary outcome cardiovascular hospitalisation or cardiovascular death showed that the addition of rosiglitazone to standard drug therapy did not improve cardiovascular risk and was associated with increased heart failure hospitalisation and a potential increase in myocardial infarction [7]. Following FDA and EMA reassessment, rosiglitazone was withdrawn from the market in 2010. Furthermore, and more generally, trials using a surrogate primary outcome have been shown to overestimate the health benefits of interventions by > 40% (adjusted ratio of odds ratios: 1.46, 95% CI: 1.05 to 2.04), compared to trials using a primary PRFO [8]. Overestimation of surrogate treatment effects also has fundamental implications for payer/reimbursement organisations such as the UK National Institute for Health and Care Excellence (NICE) and may result healthcare systems funding therapies that are not truly cost-effective [9].

Therefore, it would be expected that RCTs using a primary surrogate endpoint pay close attention to this aspect of design in their reporting, e.g. clearly stating that the primary outcome is a surrogate, outlining the rationale for its use, and providing evidence of the surrogate endpoint being on the causal pathway and its validity (e.g. meta-analysis of RCTs showing a strong association of the treatment effect on the surrogate endpoint and a PRFO [10]). Unfortunately, this appears not to be the case; the most recent analysis, a review of RCTs published in 2005 and 2006, found that 17% (107/626) used a surrogate primary endpoint and, of these, only a third discussed whether the surrogate endpoint was validated [11].

## Need for improved reporting

Implementing reporting guidelines such as the widely used SPIRIT (Standard Protocol Items: Recommendations for Interventional Trials) 2013 [12] and CONSORT (Consolidated Standards of Reporting Trials) 2010 statements [13] can improve completeness of protocol and RCT reporting [14]. However, these guidelines and their extensions, including SPIRIT-PRO [15] and CONSORT-PRO [16]) and ongoing CONSORT-Outcomes [17], do not directly address the issues of surrogate endpoint reporting.

**Fig. 1:**
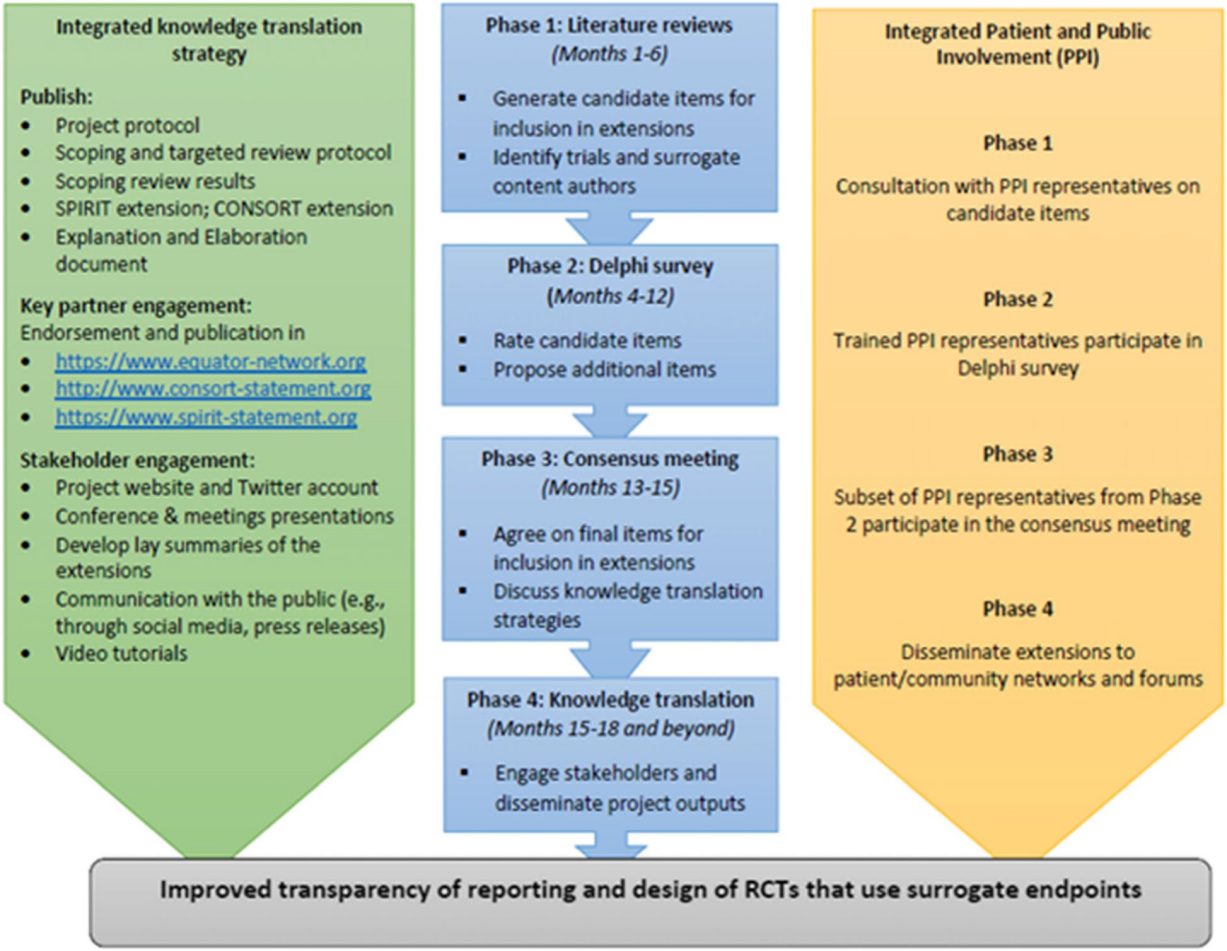
The main project phases and timings

We therefore announce a new initiative to develop guideline extensions specific to surrogate outcomes (‘SPIRIT-SURROGATE’ and ‘CONSORT-SURROGATE’). The aims of these extensions are to improve the reporting RCT protocols and reports that use a surrogate primary endpoint. These extensions will be developed following Enhancing Quality and Transparency of Health Research (EQUATOR) methodology. Figure 1 summarises the main project phases and timings.

To make these new extensions as usable as possible, we would like to invite interested individuals from various communities (trial methodologists, journal editors, healthcare industry, regulators and payers, and patient/public representative groups), particularly those with interest/experience in the use of surrogate endpoints in trials, to contribute. Journal readers can follow project progress and indicate their expression interest in participation via our project webpage (https://www.gla.ac.uk/spirit-consort-surrogate).

## Data Availability

Not applicable.

## Abbreviations

CONSORT: Consolidated Standards of Reporting Trials
EMA: European Medicines Agency
FDA: Food and Drug Administration
HbA1c: Glycosylated haemoglobin
PRFO: Patient/participant reported final outcome
RCT(s): Randomised controlled trial(s)
SPIRIT: Standard Protocol Items: Recommendations for Interventional Trials
US: United States
